# Vaccination of horses with a recombinant modified vaccinia Ankara virus (MVA) expressing African horse sickness (AHS) virus major capsid protein VP2 provides complete clinical protection against challenge

**DOI:** 10.1016/j.vaccine.2014.04.036

**Published:** 2014-06-17

**Authors:** Berta Alberca, Katarzyna Bachanek-Bankowska, Marta Cabana, Eva Calvo-Pinilla, Elisenda Viaplana, Lorraine Frost, Simon Gubbins, Alicia Urniza, Peter Mertens, Javier Castillo-Olivares

**Affiliations:** a The Pirbright Institute, Ash Road, Pirbright, Woking GU24 0NF, Surrey, UK; b Zoetis-Spain, Ctra de Comprodon, Finca La Riba, 17813 Vall de Bianya, Girona, Spain

**Keywords:** African horse sickness, Vaccine, Modified vaccinia Ankara, VP2, Protection

## Abstract

African horse sickness virus (AHSV) is an arthropod-borne pathogen that infects all species of equidae and causes high mortality in horses. Previously, a recombinant modified vaccinia Ankara (MVA) virus expressing the protein VP2 of AHSV serotype 4 was shown to induce virus neutralising antibodies in horses and protected interferon alpha receptor gene knock-out mice (IFNAR −/−) against virulent AHSV challenge.

This study builds on the previous work, examining the protective efficacy of MVA-VP2 vaccination in the natural host of AHSV infection. A study group of 4 horses was vaccinated twice with a recombinant MVA virus expressing the major capsid protein (VP2) of AHSV serotype 9. Vaccinated animals and a control group of unvaccinated horses were then challenged with a virulent strain of AHSV-9. The vaccinated animals were completely protected against clinical disease and also against viraemia as measured by standard end-point dilution assays. In contrast, all control horses presented viraemia after challenge and succumbed to the infection.

These results demonstrate the potential of recombinant MVA viruses expressing the outer capsid VP2 of AHSV as a protective vaccine against AHSV infection in the field.

## Highlights

• A recombinant modified Vaccinia Ankara virus expressing VP2 of African horse sickness virus serotype 9 was generated.
• Four horses were vaccinated on days 0 and 20. Three unvaccinated controls were used.
• Vaccinated and control horses were challenged intravenously with 10^7.4^TCID50 of AHSV-9 on day 34 of the study.
• At challenge, vaccinates had virus neutralising antibodies but were negative for antibodies to AHSV-VP7.
• All vaccinates were completely protected against clinical signs of African horse sickness.

## 1 Introduction

African horse sickness (AHS) is a lethal arboviral disease of equids with mortality rates that can exceed 95% in susceptible populations. The disease is endemic to sub-Saharan Africa but sporadically escapes from this geographical area and extends to the North of Africa, the Middle East, the Arabian Peninsula, India and Pakistan. In the past, the disease has also spread to Europe, specifically to Spain in 1969 and Spain and Portugal in 1987 [1,2]. The latest outbreak in Western Mediterranean countries lasted 5 years [3,4].

To date no effective treatment exists for AHS and consequently control of the disease relies on preventive vaccination. AHS vaccines, based on attenuated AHS viruses, have been in use in South Africa for almost 100 years and permitted the subsistence of horses in that part of the world. There are nine different serotypes of AHS virus (AHSV) and protective immunity is long-lived against homologous serotypes. Thus, vaccination in endemic countries is normally performed by administration of combinations of representative attenuated strains of each of the virus serotypes. Serotypes 5 and 9 are normally excluded from vaccine formulations. Serotype 5 is difficult to attenuate and partially cross-reacts with serotype 8; and serotype 9 does not normally occur in South Africa (the main AHSV vaccine manufacturing country) and partially cross-reacts with serotype 6 [3,5,6].

Despite their apparent efficacy, live AHSV vaccines have a number of disadvantages [4]. These include: (a) the risk of reversion to virulence; (b) the risk of gene segment re-assortment between field and vaccine strains; (c) the risk of introducing foreign topotypes into a new geographical region, since vaccines are based on South African strains; (d) the absence of DIVA (Differentiating Infected from Vaccinated Animals) capacity, that is the inability to serologically differentiate vaccine-induced immunity from that induced by natural infection; and (e) the contra-indications for use in pregnant mares because of their teratogenicity. In addition to these science-based shortcomings of the live vaccines it is also important to consider the potential logistical delays between the first detection of an outbreak and the deployment of sufficient vaccine doses to where they would be needed.

The recognised shortcomings of existing live AHSV vaccines has meant that alternative vaccination strategies have been pursued over the years. These have included the use of killed vaccines [7–9], vaccines based on baculovirus-expressed AHSV capsid proteins [10], DNA vaccines [11] and those based on the use of poxvirus expression vectors [12–14]. The latter appear to be a particularly promising strategy, which has started to produce encouraging results. We have demonstrated recently that recombinant MVA viruses expressing VP2 from AHSV serotype 4 (MVA-VP2), the major capsid protein of AHSV and main target of virus neutralising antibodies (VNAb), induced VNAb in horses and complete protection against virulent challenge in a mouse model [12,13].

These studies have now been extended to determine the protective capacity of MVA-VP2 vaccination against AHSV clinical disease and viraemia in the target species, the horse.

## 2 Materials and methods

### 2.1 Viruses

A recombinant MVA expressing the VP2 protein of the AHSV-9 reference strain (PAKrrah/09), was generated using standard published techniques [12,13,15] using primary chicken embryo fibroblasts (CEF), obtained from the Microbiological Services of the Pirbright Institute (MSPI). This virus was designated MVA-VP2(9). The DF-1 cell line [16], obtained from MSPI and currently available from the ATCC (CRL-12203) was used to grow the MVA-VP2(9) virus, with an input multiplicity of infection (moi) of 0.1. When maximum cytopathic effect (cpe) had been reached, the supernatant media and cell debris were harvested and centrifuged at 930 × *g*, 4 °C. The low titre supernatant was discarded and the highly infective pellet was re-suspended in Dulbecco's Modified Eagle's Essential Medium (DMEM) supplemented with penicillin-streptomycin. The re-suspended pellet was titrated, stored at −70 °C, and used for vaccination after being diluted in DMEM.

The AHSV-9 challenge virus used was from the Orbivirus Reference Collection at Pirbright. It was a derivative of the AHSV-9 strain KEN2006/01, a field isolate collected from a dead foal in Nairobi in 2006. The virus was grown in Culicoides KC cells, titrated in Vero cells by a standard end-point dilution assay, and subsequently passaged in Vero cells. The final titre of the virus, expressed as 50% Tissue Culture Infective Dose (TCID_50_) per ml, was 10^6.8^

### 2.2 Horses

For the study, a mixture of seven male and female cross-breed horses of 1 year of age were used. The animals were randomly assigned to two different groups. Four were vaccinated with MVA-VP2(9) and three animals acted as non-vaccinated controls. Before vaccination, horses were group housed outdoors for a quarantine period. During this period, routine veterinary health checks were performed. One week before vaccination, the animals were moved to the experimental facilities for acclimatization to the new environment. All sampling procedures and clinical examinations of the animals were performed by an experienced veterinary surgeon. Trained animal husbandry technicians were responsible for day-to-day husbandry procedures.

This study was approved with the authorization number 339 by the local Ethical Review Committee of Zoetis, Olot, Spain, in compliance with national guidelines and EU regulations for projects using animals for research purposes. The facilities and husbandry procedures complied with the EU Directive 2010/63/EU.

### 2.3 Vaccination and challenge

Three animals were not vaccinated and acted as controls. The remaining four horses received the MVA-VP2 (9) vaccine, with vaccine dose (10^8^ pfu/ml) being split into an intramuscular (0.5 ml) and a subcutaneous (0.5 ml) injection, both given on the side of the neck. Vaccination was on day 0 (V1), with a booster being administered on day 20 (V2). The animals were challenged 14 days after the last vaccination by intravenous injection (4 ml) with a challenge dose of 10^7.4^ TCID_50_ per horse. Horses were clinically examined twice a day following infection with AHSV-9 and more often once clinical signs began to develop. Clinical signs and rectal temperatures were recorded. Humane end-points established for this experiment included any of the following: presence of severe generalised oedema, severe dyspnoea, presence of foamy nasal exudate, severe depression with prostration or high rectal temperatures (above 40 °C) for four consecutive days.

### 2.4 Sample collection

Blood samples for serological analysis were collected on days 0 (V1), 6, 13, 20 (V2), 27 and 34 (challenge). Blood samples for virus isolation and RT-PCR were collected on day 34 (challenge day) and days 3, 7, 9, 11, 14, 17 and 21 post-challenge.

### 2.5 Serology

To measure VNAb, serum samples were first inactivated at 56 °C for 30 min and then titrated in a 96-well flat-bottomed tissue culture plate. Standard published methods were followed [13,17]. Briefly, each dilution was incubated with 100 TCID_50_ of the AHSV-9 virus, incubated for 4 days and end-points defined as the dilution that neutralised virus infectivity of 50% of the replicates. Titres were calculated according to Karber [18].

Serum samples were also analysed using a VP7 ELISA test to determine the antibody responses specific for this AHSV antigen. The INGEZIM VP7 ELISA (Ingenasa, Madrid, Spain) was used according to the manufacturer's protocol.

### 2.6 Virus isolation and real time RT-PCR

Blood samples were processed as previously described [12]. The treated samples were serially diluted, in triplicate, on a micro titre plate and each sample incubated with 100 μl/well of a cell suspension containing 10^5^ Vero cells/ml. After 4 days incubation the highest sample dilution causing cytopathic effect in 50% of the replicate wells was recorded and the Tissue Culture Infectious Dose 50 (TCID_50_) of the sample calculated according to Karber: Log_10_TCID50 = *a* − *D* (*Sp* − 0.5), where *a* is the log_10_ of highest dilution giving 100% cpe; *D* is the log_10_ of the dilution factor; *Sp* is the sum of the fractions of cpe-positive replicates; and 0.5 is a constant. The final viraemia results were expressed as TCID_50_/ml of blood.

Real time RT-PCR was performed according to published procedures [19]. Briefly, viral RNA was extracted from blood samples using the BioSprnt 96 DNA Blood kit (QIAGEN) following manufacturer's instructions. A known concentration of a synthetic double-stranded RNA from the viral RNA segment encoding VP7 was used as a standard to quantify the viral genome copies. This synthetic double stranded RNA was generated using a pMA plasmid (Life Technologies) coding for a 107 bp fragment from AHSV-VP7 gene flanked on both sides by T7 polymerase promoters. For the generation of the double-stranded RNA (dsRNA), both RNA strands were transcribed in vitro using the MEGAshortscript™T7 Kit (Ambion) following manufacturer instructions. Transcribed RNAs were purified using the MEGAclear™ kit (Ambion), checked by agarose gel electrophoresis and concentration determined by spectrophotometry. Transcribed ssRNA molecules were mixed in precise equimolar amounts. This dsRNA was adjusted to 7.2 × 10^7^ copies/μl. Serial ten-fold dilutions of the standard RNA were included in each assay. Cycle Threshold (Ct) values were plotted against the serial dilutions of the standard RNA to produce the standard curve to determine the genome copies per ml of blood sample.

## 3 Results

### 3.1 Immunogenicity of MVA-VP2(9)

All horses were sero-negative at the beginning of the study and developed serum VNAb upon inoculation with MVA-VP2(9). No adverse reactions to vaccination were seen, other than a transient inflammation at the injection site which subdued after 24 h. On day 34 of the study, the vaccinated horses and 3 unvaccinated controls were challenged with AHSV-9.

### 3.2 Clinical signs and pathology

**Fig. 1:**
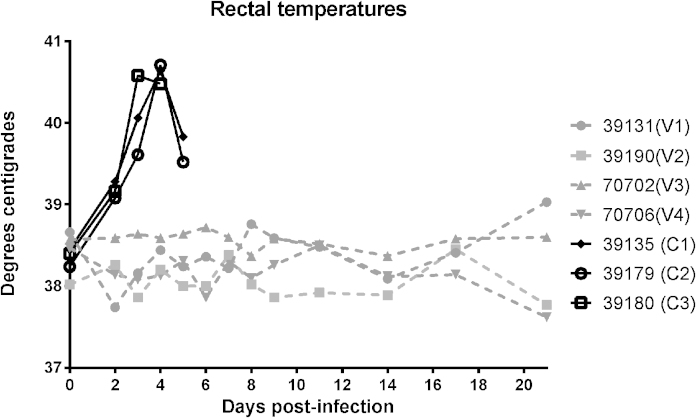
Individual rectal temperatures of vaccinated (V1–V4) and control (C1–C3) horses recorded over the whole study period.

Following challenge with AHSV-9, all vaccinated animals remained clinically normal and their rectal temperatures remained within physiological ranges until the end of the study (Fig. 1). In contrast, all the control horses developed clinical signs consistent with the cardiac form of African horse sickness. They became febrile by day 2 post-infection as rectal temperatures reached values ranging between 39.08 to 39.28, a significant rise compared with the vaccinated group (Wilcoxon rank sum test: *P* = 0.05). These temperatures peaked on day 3 (horse C3) and day 4 (horses C1 and C2), and then declined in the hours before death. Clinical signs in the control animals were present by day 3 post-infection and comprised: mild general malaise and depression; palpebral oedema and conjunctivitis; and mild nasal discharges. These clinical signs slightly worsened on day 4 and progressed very rapidly thereafter. The three control horses died between the end of day 5 (C3) and day 6 (C1 and C2).

The post-mortem lesions of control horses were consistent with the cardiac form of AHS, and included: oedema, congestion and haemorrhages of the ocular conjunctiva; the presence of a yellow gelatinous oedema in the inter-muscular fasciae of the neck and sub-scapular region, oesophagus and epicardial surfaces; hydropericardium; hydrothorax; sub-endocardial haemorrhages; and congestion of the kidneys, liver, spleen and stomach mucosa. The lungs presented mildly enlarged interlobular septi but the typical frothy fluid of the ‘pulmonary form’ of AHS was not present.

### 3.3 Viraemia and real time RT-PCR

The results of these tests are presented in Tables 1 and 2. All vaccinated animals were negative for infectious virus in blood whereas the control horses developed viraemia with viral titres that ranged between 10^4.5^ to 10^4.6^ TCID_50_/ml on day 3, and between 10^5.5^ to 10^5.8^ TCID_50_/ml on day 5. The differences between vaccinates and controls on each day were statistically significant (Wilcoxon rank-sum test: *P* = 0.03 for both days)

Real time RT-PCR results indicate that there were significant differences in the viral load between vaccinates and controls. The mean viral RNA log_10_ copy number on day 3 was 10^6.8^ for controls and 10^2.9^ for vaccinated animals (Wilcoxon rank-sum test *P* = 0.06), while on day 5 it was 10^7.8^ for controls and 10^1.6^ for vaccinated animals (Wilcoxon rank-sum test *P* = 0.05). The vaccinated animals remained positive by RT-PCR on subsequent days post-challenge and some animals that were negative produced a positive result on later samples. By day 21, vaccinated horses were still positive by RT-PCR although infectious virus was undetectable by the end-point dilution assay.

### 3.4 Antibody responses following MVA-VP2(9) vaccination and AHSV-9 challenge

As expected, all four animals vaccinated with MVA-VP2(9) developed VNAb by the time of challenge with titres ranging between 1.6 to 2.4 (Table 3). Following AHSV-9 challenge these VNAb titres increased more than four-fold in all four animals and the final titres recorded on day 28 post-challenge reached values of between 2.3 to more than 3.1. All non-vaccinated control horses were negative for VNAb at virus challenge and did not develop VNAb before they succumbed to AHSV-9 infection.

Antibodies to AHSV-VP7 were detected in serum samples of the vaccinated horses only after challenge (Table 4). As expected all horses were negative by the VP-7 ELISA test on the day of challenge (day 34).

## 4 Discussion

This study in the disease relevant host, the horse, was aimed at determining the protective capacity of vaccines based onMVA-VP2 against virulent AHSV challenge. This work focused on AHSV-9. Thus, the MVA-VP2(9) recombinant vaccine was constructed using the genome segment encoding VP2 from the AHSV-9 reference strain (PAKrrah/09) and vaccinated animals were challenged with the AHSV-9 strain KEN/2006/01.

Ponies immunised with MVA-VP2(4) in a previous study [13] and those vaccinated with MVA-VP2(9) in this study developed VNAb titres after two doses and reached titres against homologous virus, ranging between 1.8 to 1.9 or between 1.6 to 2.4, respectively. These results are in line with studies by others using poxvirus vectors expressing AHSV-VP2. Thus, horses vaccinated with 10^7.1^ TCID_50_ of a canarypox-based AHSV vaccine [14] expressing VP2 and VP5 developed serum VNAb titres of 20–40 (1.3–1.6 log_10_); and use of a recombinant vaccinia virus (strain WR) expressing AHSV-4 VP2 also induced VNAb in horses [20], albeit at low titres and only after 3 vaccine inoculations.

In this study, vaccination of horses with MVA-VP2(9) showed very high levels of protection despite the high challenge virus dose used. Clinical signs were completely absent in vaccinates and the rectal temperatures were within normal physiological ranges during the study period. In contrast, the control horses experienced a peracute AHSV cardiac syndrome accompanied by high rectal temperatures. Vaccinated animals were also completely protected against viraemia as measured by a standard end-point dilution assay demonstrating the potential of MVA-VP2 vaccination to prevent onward transmission by the insect vectors. These levels of protection are in line with those of other vaccination approaches [6,14], although the mechanisms of immunity through which vaccine protection was exerted might be different. The current live attenuated vaccines induce a low VNAb titre in vaccinates after a primary vaccination course suggesting cell-mediated immunity plays an important role in clearance of AHSV infection in horses vaccinated with live attenuated or canarypox VP2/VP5 vaccines [6,14,21]. In the mouse model both cell-mediated and VNAb responses were stimulated by MVA-VP2 vaccination, however passive transfer experiments have shown that humoral immunity plays a critical role in protection against AHSV [12,22]. In the present study, MVA-VP2 vaccination induced a relatively high VNAb titre compared to that induced by existing live attenuated vaccines, but cell-mediated immune responses have not yet been measured.

In this study we have detected the presence of viral RNA, though at lower levels than in the control animals, in non-infectious blood samples from the vaccinated horses for up to day 21 post-challenge. The high virus challenge dose (10^7.4^ per horse) given by the intravenous route, the natural capacity of AHSV to bind erythrocytes [23] and the high sensitivity of RT-PCR techniques could explain the presence of viral RNA in the non-infectious blood of vaccinated horses. This is consistent with the findings obtained during the development of an RT-PCR diagnostic assay of AHSV in which viral RNA was detected from the blood of horses inoculated intravenously with 10^5.5^ TCID_50_/ml up to day 97 post-infection [24]. It is very difficult to discern from our data whether AHSV RNA in the vaccinates was a result of viral replication in the host or not. Analysis of the antibody responses by the virus neutralisation test and by the VP7 ELISA test showed more than a four-fold increase in VNAb titre and an increase in VP7 ELISA antibody levels in paired serum samples collected at day 34 (challenge day) and day 62. This could be an indication of a low level of viral replication in the vaccinates but this could also be the result of an anamnestic response of immune animals to re-exposure to an AHSV antigenic stimulus. Alternatively, virus particles neutralised by serum antibodies, could still be circulating in the vaccinates and could have been the source of viral RNA detected by the RT-PCR assay. Further work is needed to elucidate whether MVA-VP2 vaccination induces a complete sterile immunity but from the results of our study this immune response was sufficient to abrogate AHSV infectivity and to prevent any clinical disease and pyrexia in horses challenged with a high dose of AHSV.

This study has demonstrated that MVA vaccines expressing VP2 alone are capable of inducing protective immunity, showing that co-expression of VP5 or other capsid proteins is not essential for the induction of a protective response. Other studies using AHSV-VP2 based vaccines, using baculovirus expression, plasmid DNA or replication-competent vaccinia virus vectors, showed that VP2 alone can induce VNAb and protective immunity in horses [11,20,25]. However, other studies showed that co-expression of VP5 seemed to improve immunogenicity of VP2-based recombinant vaccines [14,26]. It is possible therefore, that co-expression of VP2 and VP5 from the same MVA recombinant vaccine vector results in improved immunogenicity.

The MVA-VP2 vaccination approach has worked with AHSV serotypes 4 and 9, and other recombinants expressing the AHSV-VP2 from other serotypes can be easily constructed to generate the complete set of monovalent AHSV vaccines based on MVA.

AHS is a lethal disease of horses that currently causes severe animal and economic loses in Africa and has the capacity to spread to Europe, as has been seen with bluetongue in the recent past. The primary way of controlling this disease currently is by the use of the live attenuated vaccines, which are regarded as unsuitable for non-endemic countries for biosafety reasons. Our results indicate that the MVA-VP2 vaccine strategy is highly protective, and is compatible with a DIVA (differentiation of infected against vaccinated animals) strategy. This feature would prevent the spread of AHSV outbreaks in non-endemic countries without compromising sero-surveillance and would enable a ‘vaccination to live’ policy to be adopted as the vaccine allows for the demonstration of disease-free status by serological discriminatory diagnostic tests (VP7 ELISA). In our study, we used the VP7 ELISA, the Office Internatinal des Epizooties (OIE) prescribed serological test for international trade, and showed that infection of MVA-VP2 vaccinated animals could be detected by using this assay, showing that horses within an AHSV-risk area could potentially be vaccinated with MVA-VP2 and the spread of AHSV infection could still be tracked by serological screening of vaccinated animals. In addition, MVA-VP2 vaccination could also be used in endemic countries to control AHS since it could prevent disease and transmission and would facilitate, due to its differential diagnostic capability, the movement of equids between different AHSV controlled geographical regions. The use of this DIVA compatible vaccination approach could also facilitate international trade of horses from the African continent.

In conclusion, we have demonstrated the potential of MVA-VP2 vaccination as a valid strategy for the prevention of AHS. The results obtained are very encouraging and the prospects of using a vaccine that is protective, safe and effective and that can be used both in endemic and non-endemic areas deserve further investigation.

## Figures and Tables

**Table 1:** Viraemia- end-point dilution assay: results of the microtitrevirus plaque assays performed on blood samples collected from the vaccinated and control horses following challenge with AHSV-9. The results are expressed as (log_10_TCID_50_/ ml of blood) of individual samples.

Group	Horse	Day 0	Day 3	Day 5	Day 7	Day 9	Day 11	Day 14	Day 17	Day 21
Vaccine MVA-VP2 (9)	V1	–	–	–	–	–	–	–	–	–
	V2	–	–	–	–	–	–	–	–	–
	V3	–	–	–	–	–	–	–	–	–
	V4	–	–	–	–	–	–	–	–	–
Controls	C1	–	4.6	5.5	ns*	ns	ns	ns	ns	ns
	C2	–	4.5	5.8	ns	ns	ns	ns	ns	ns
	C3	–	4.5	5.8	ns	ns	ns	ns	ns	ns

–: Negative result.

* ns: No sample.

**Table 2:** Viraemia—RT-PCR: results of the RT-PCR assay for the detection of viral RNA from blood samples collected from vaccinated and control horses challenged with AHSV-9. The results are expressed as RNA copy numbers (log_10_) per ml of blood of individual samples.

Group	Horse	Day 0	Day 3	Day 5	Day 7	Day 9	Day 11	Day 14	Day 17	Day 21
Vaccine MVA-VP2 (9)	V1	–	2.73	2.82	3.44	3.76	2.92	4.83	5.41	5.16
	V2	–	2.72	–	–	–	3.04	3.6	4.01	3.09
	V3	–	3.09	–	–	5.78	–	2.7	2.66	2.81
	V4	–	2.97	3.51	4.03	5.29	6.21	6.1	5.63	4.93
Controls	C1	–	6.95	7.76						
	C2	–	6.38	7.81						
	C3	–	7.07	7.94						

–: Negative result.

**Table 3:** VNAb titres (log_10_) of serum samples collected from vaccinated and control horses.

Group	Horse	Day 0	Day 34 (challenge)	Day 62
Vaccine MVA-VP2(9)	V1	−	1.6	2.3
	V2	−	2.4	>3.1
	V3	−	1.8	2.68
	V4	−	1.8	>3.1
Controls	C1	−	−	*ns
	C2	−	−	ns
	C3	−	−	ns

−: Negative result.

* ns: No sample.

**Table 4:** VP7-specific antibody levels, measured by the VP-7 ELISA test, of serum samples collected from vaccinated and control horses. Figures represent the percentage of antibody binding inhibition of a VP7-specific antibody to the VP7 antigen for each of the serum samples analysed. Positive VP-7 specific antibody levels are indicated in bold.

Group	Horse	Day 0	Day 34 (challenge)	Day 62
Vaccine MVA-VP2(9)	V1	Not done	37.20	**55.76**
	V2	Not done	23.69	**79.43**
	V3	Not done	27.13	**65.52**
	V4	Not done	29.43	**76.10**
Controls (AHV9)	C1	Not done	29.68	*ns
	C2	Not done	32.66	ns
	C3	Not done	29.31	ns

* ns: No sample.
